# Intelligent Estimation of Exercise Induced Energy Expenditure Including Excess Post-Exercise Oxygen Consumption (EPOC) with Different Exercise Intensity

**DOI:** 10.3390/s23229235

**Published:** 2023-11-16

**Authors:** Junhyung Moon, Minsuk Oh, Soljee Kim, Kyoungwoo Lee, Junga Lee, Yoonkyung Song, Justin Y. Jeon

**Affiliations:** 1 Department of Computer Science, Yonsei University, 50 Yonsei-ro, Seodaemun-gu, Seoul 03722, Republic of Korea; jh.moon.cs@yonsei.ac.kr (J.M.); soljee.kim@yonsei.ac.kr (S.K.); kyoungwoo.lee@yonsei.ac.kr (K.L.); 2 Department of Sport Industry Studies, Yonsei University, 50 Yonsei-ro, Seodaemun-gu, Seoul 03722, Republic of Korea; minsuk_oh@yonsei.ac.kr (M.O.); lapine1019@yonsei.ac.kr (Y.S.); 3 Frontier Research Institute of Convergence Sports Science, 50 Yonsei-ro, Seodaemun-gu, Seoul 03722, Republic of Korea; 4 Graduate School of Sport Science, Kyung Hee University, 1732 Deogyeong-daero, Giheung-gu, Yongin-si 17104, Republic of Korea; jalee@khu.ac.kr; 5 Exercise Medicine Center for Diabetes and Cancer Patients (ICONS), 50 Yonsei-ro, Seodaemun-gu, Seoul 03722, Republic of Korea

**Keywords:** energy expenditure, excess post-exercise oxygen consumption, heart rate, machine learning

## Abstract

The limited availability of calorimetry systems for estimating human energy expenditure (EE) while conducting exercise has prompted the development of wearable sensors utilizing readily accessible methods. We designed an energy expenditure estimation method which considers the energy consumed during the exercise, as well as the excess post-exercise oxygen consumption (EPOC) using machine learning algorithms. Thirty-two healthy adults (mean age = 28.2 years; 11 females) participated in 20 min of aerobic exercise sessions (low intensity = 40% of maximal oxygen uptake [VO${}_{2}$ max], high intensity = 70% of VO${}_{2}$ max). The physical characteristics, exercise intensity, and the heart rate data monitored from the beginning of the exercise sessions to where the participants’ metabolic rate returned to an idle state were used in the EE estimation models. Our proposed estimation shows up to 0.976 correlation between estimated energy expenditure and ground truth (root mean square error: 0.624 kcal/min). In conclusion, our study introduces a highly accurate method for estimating human energy expenditure during exercise using wearable sensors and machine learning. The achieved correlation up to 0.976 with ground truth values underscores its potential for widespread use in fitness, healthcare, and sports performance monitoring.

## 1. Introduction

Energy expenditure (EE) serves as a vital metric reflecting the extent of physical activity and plays a pivotal role in the formulation of exercise prescriptions. Consequently, achieving accurate measurements and estimations of EE is of paramount importance. The doubly labelled water method and other indirect calorimetry methods are the gold standards for EE estimation [1]. However, due to their limited availability, there has been a rise in wearable sensors that use accessible methods such as accelerometry, barometry, and physiological measurement [2,3,4,5,6,7,8]. Additionally, measuring heart rate, a well-established health indicator, has proven effective in estimating EE because of its strong correlation with it [9,10,11,12].

Unfortunately, traditional EE estimation models have overlooked the excessive post-exercise oxygen consumption (EPOC) rate. EPOC is an important variable that exhibits the increase in EE quantified by oxygen consumption following exercise activity, and EPOC is highly variable depending on the type, intensity, and/or duration of exercise [13,14,15,16,17]. While a previous study [18] examined a heart rate sensor to estimate EPOC, it faced challenges due to the weak correlation between heart rate and EPOC, and it lacked EE data during exercise. Recent studies have also explored portable sensors as alternatives to indirect calorimetry systems for EE estimation [19,20]. However, these studies did not address the estimation of EPOC. In addition, variations in individuals’ health characteristics can result in varied levels of EE during exercising, affecting the accuracy of EE estimation [3]. Therefore, additional work is warranted using an innovative technique to accurately estimate EE while considering both EPOC and individual physical characteristics.

Previously, we introduced a unique methodology using machine learning algorithms to estimate EE, specifically considering EPOC [21]. We observed variations in EE that were affected by both exercise intensity and physical characteristics of the study participants. Based on the observations, we anticipated that considering these affecting factors in the model design phase could increase EE estimation accuracy. In addition, exploring design space in machine learning by incorporating additional features and regulating multiple parameters could improve EE estimation. As a result, in this work, we crafted an advanced EE estimation model for exercise, leveraging in-depth machine learning analysis and accounting for parameters like EPOC. The objectives of this study were (1) to examine the trends of consumed energy during and after aerobic exercise and (2) to estimate EE across different conditions of physical characteristics and exercise intensities.

## 2. Materials and Methods

### 2.1. Study Protocols and Participants

We used our data collected from a designed laboratory experiment comprising preparation (PRE), exercise with low intensity (EXER_low_) or exercise with high intensity (EXER_high_), and rest (REST), as presented in our previous work [21]. We obtained consents from all participants and instructed them not to eat for 8 h preceding their scheduled study visits. In the PRE phase, we measured participants’ height and weight using an electric extensometer (BSM 340, Biospace, Seoul, Republic of Korea), rounding to the nearest 0.1 cm and 0.1 kg, respectively.

Body mass index (BMI) was calculated as the weight divided by the square of height (kg/m${}^{2}$). Participants then engaged in 20-min walking or running sessions on a motorized treadmill (Q-stress, TM55, Quinton, VA, USA) at one or both of low and high intensity. To ensure a tailored and accurate measurement of exercise intensity, we assessed maximal oxygen uptake (VO${}_{2}$ max) for each participant. During the exercise sessions, participants were monitored with a digital heart rate monitor (Polar H7, Polar Electro, Kempele, Finland) to ensure that each was exercising at the correct intensity—low (40% of VO${}_{2}$ max) and high (70% of VO${}_{2}$ max)—relative to their individual cardiorespiratory fitness levels. This approach allowed us to standardize the exertion level across participants, accommodating individual differences and ensuring the comparability of our results. After the exercise sessions, participants lay in a supine position until their metabolic rate returned to a resting state (REST). We used an automatic gas analyzer (Respina 1H 26, NEC San-Ei, Tokyo, Japan) to measure EE through breath-by-breath analysis, providing a ground truth reference. The entire experiments were approved by the Institutional Review Board of Yonsei University (1040917-201411HRBR-249-02).

A total of 32 healthy participants completed the experiments, each completing the protocol only once. The sample size was determined by referencing a relevant systematic review study [22], in which EPOC was the primary outcome variable. To calculate our sample size, we utilized a one-sample α level of 0.05, a power of 0.80 and an effect size (Cohen’s d value) of 0.5. This calculation led to a final sample size of at least 27 participants. Among the 32 participants, 25 completed both EXER_high_ and EXER_low_ (50 experiments). The remaining seven participants completed either EXER_high_ or EXER_low_ (seven experiments). Additionally, three (P1–P3) out of the 25 participants engaged in extra experiments (seven experiments) of which the duration was configured as 10 or 30 min (P1: 30 m EXER_low_, P2: 30 m EXER_high_ and 30 m EXER_low_, P3: 10 and 30 m EXER_high_ and 10 and 30 m EXER_low_). In summary, the 32 participants collectively completed 64 experiments, amounting to 14,270 min in total.

### 2.2. Feature Extraction and Model Design

To design EE estimation models using machine learning algorithms, we extracted useful features from the collected data which are related to EE. We calculated features of heart rate (F_heart_), physical information (F_physical_), and exercise information (F_exercise_) as shown in Table 1. Before we calculated F_heart_ features, we normalized all heart rate values of each experiment. Building on our previous work [21], we introduced additional features like EP_T and EX_or_EP to distinctly represent exercise and resting periods. For the unit size of feature extraction, most existing works have used the entire exercise period to estimate corresponding EE. However, examining subperiods can effectively capture dynamic changes of EE within shorter time frames. Accordingly, in this work, we configured the estimation window size from 5 to 20 min increasing by 1 min with the overlapping approach. In total, we extracted 16 features, some of which are new, for different EE estimation window sizes.

Using the extracted features, we designed EE estimation models with 10 machine learning algorithms from five categories supported in Weka software (version 3.7.13) [23]. They are LinearRegression (LR) and MLPRegressor (MLPR) from *functions*, IBk and LWL from *lazy*, AdditiveRegression (AR) and RegressionByDiscretization from *meta*, DecisionTable (DT) and M5Rules (M5) from *rules*, and RandomForest and REPTree from *trees*. In this work, we additionally used a neural network regressor implemented using Python with TensorFlow library.

For all the algorithms, we exploited 10-fold cross validation.

### 2.3. Data Analysis

To analyze energy expenditure trends based on physical characteristics and exercise intensities, we used data of 25 EXER_high_ and 25 EXER_low_ experiments out of the 64 ones mentioned in Section 2.1. We first categorized the data based on exercise intensity (EXER_high_ and EXER_low_), gender (Male and Female), age groups (10–20 s and 30–40 s), and BMI ranges (BMI ≥ 23 and BMI < 23). For BMI-based classification, we referred to the standard determining the overweight state for the Asian population [24]. To investigate the impact of different exercise durations on EE, we examined data of P1, P2, and P3 introduced in Section 2.1.

For machine learning-based EE estimation model design, we used data from all 64 experiments. We established a conventional method (Conv.) as a baseline to compare with our machine learning-based EE estimation models. Conventional works have utilized data collected during exercise to estimate EE introduced by the exercise. They aggregated the collected data from the entire exercise period into representative indices such as the average. Accordingly, the Conv. in this paper is a method to estimate EE of each exercise by using aggregated heart rate data for the entire exercise period. We evaluated the performance of our models against the baseline using the Pearson Correlation Coefficient (PCC) [25], defined in Equation (1), and the root mean square error (RMSE), presented in Equation (2). RMSE is the most commonly used metric in EE estimation field [26]. In Equations (1) and (2), *E*[*] is the expected value of *, μ is the mean, σ is the standard deviation, *X* is the estimated EE, *Y* is the ground truth of EE, *N* is the number of instances, and *time* is the unit size of the estimation window in minutes.

(1) $$PCC=\frac{E[\left(X-\mu_{X}\right)\left(Y-\mu_{Y}\right)]}{\sigma_{X}\sigma_{Y}}$$

(2) $$RMSE\;\left(kcal/minute\right)=\frac{\sqrt{\frac{1}{N}\sum_{i=1}^{N}(X_{i}-Y_{i}{)}^{2}}}{time}$$

## 3. Results

### 3.1. Energy Expenditure Including EPOC and Physical Characteristics

Table 2 summarizes the age, height, weight, and BMI of our 32 participants, comprising 21 males and 11 females. In this study, EE_exercise_ denotes energy expenditure during exercise and EE_total_ is the sum of EE_exercise_ and EPOC. Table 3 presents the average values for age, BMI, exercise duration, EE_exercise_, REST duration, and EPOC across 50 EXER_all_ experiments (25 EXERhigh and 25 EXERlow) under various conditions. Notably, as shown in Table 3, EXER_high_, Male, 10–20 s, and BMI >= 23 generally exhibit higher average EE_exercise_ and EPOC values compared to their respective counterparts. Among these comparisons, EE_exercise_ of EXER_high_ exceeds that of EXER_low_ by the largest margin of 105.4 kcal.

Similarly, Table 4 and Table 5 categorize the data into 25 EXER_high_ and 25 EXER_low_ experiments. When comparing average EE values across conditions, the disparities are more evident in EXER_high_ than in EXER_low_. For instance, the average EE_total_ difference between Male and Female in EXER_high_ is approximately 69.0 kcal, whereas in EXERlow, it is about 49.9 kcal. While the average EE_exercise_ trends remain consistent across exercise intensities, EPOC trends vary based on the intensity. For example, the average EE_exercise_ in 10–20 s is larger than that in 30–40 s for both EXER_high_ (218.2 kcal > 180.1 kcal) and EXER_low_ (106.4 kcal > 83.0 kcal). However, the 10–20 s category has a larger average EPOC (89.3 kcal) than 30–40 s (61.2 kcal) in EXER_high_, while average EPOC appears smaller in 10–20 s (40.6 kcal) than 30–40 s (48.0 kcal) in EXER_low_. In summary, EE shows different trends according to different exercise intensities and physical characteristics.

Our observations also highlighted the influence of exercise duration on EE. P1, P2, and P3 all exhibited increased EE with longer exercise durations, irrespective of exercise intensity. Intriguingly, all three participants had a higher EPOC after a 20-min EXERlow session than after a 30-min one. P2 mirrored this trend in EXERhigh. Specifically, P3’s EPOC was highest after a 20-min EXER_low_ session compared to 10- and 30-min sessions, while the 30-min EXER_high_ session yielded the highest EPOC for P3.

**Figure 1:**
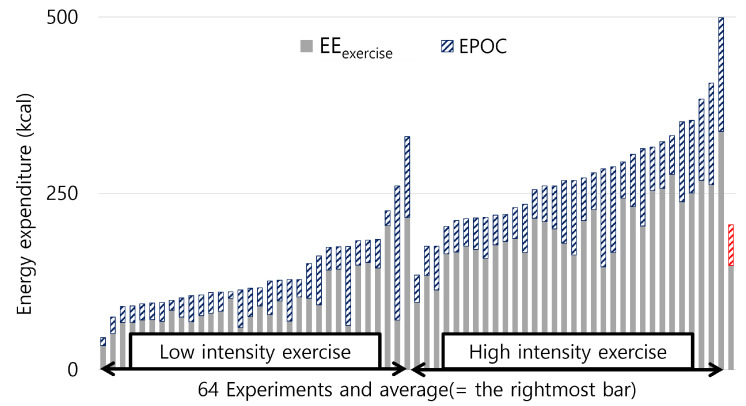
Ratio of EE_exercise_ and EPOC, respectively within EE_total_ for 64 experiments.

Figure 1 delineates EE_exercise_ (solid) and EPOC (patterned) within EE_total_ for all 64 experiments. On average, as depicted in the rightmost bar of Figure 1, EPOC constitutes 27.6% of EEtotal. The average EPOC is 57.4 kcal out of an average EE_total_ (205.3 kcal). In EXER_low_, EPOC accounts for up to 73.1% of EE_total_, while in EXER_high_, it is up to 48.9%. This indicates that neglecting EPOC in EE estimations can lead to significant inaccuracies. Therefore, for precise exercise EE estimations, it is crucial to consider both EPOC and participant attributes like gender, age, and BMI.

### 3.2. Overall EE Estimation Results Considering EPOC

**Figure 2:**
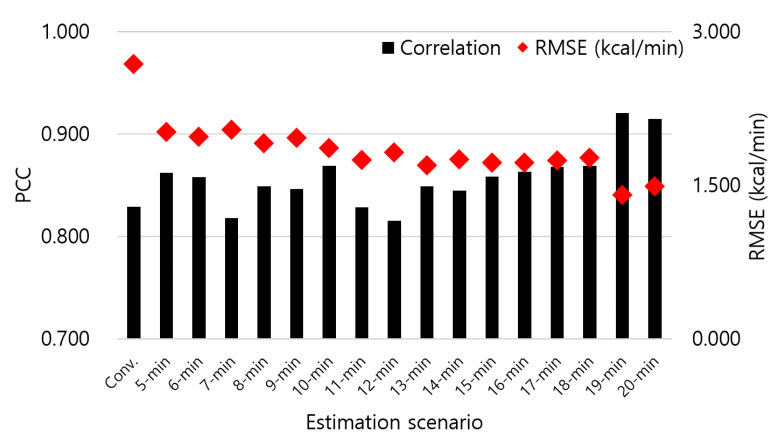
EE estimation results in terms of correlation (PCC) and error (RMSE) for a conventional method (Conv.) and our proposed method with different window sizes.

**Figure 3:**
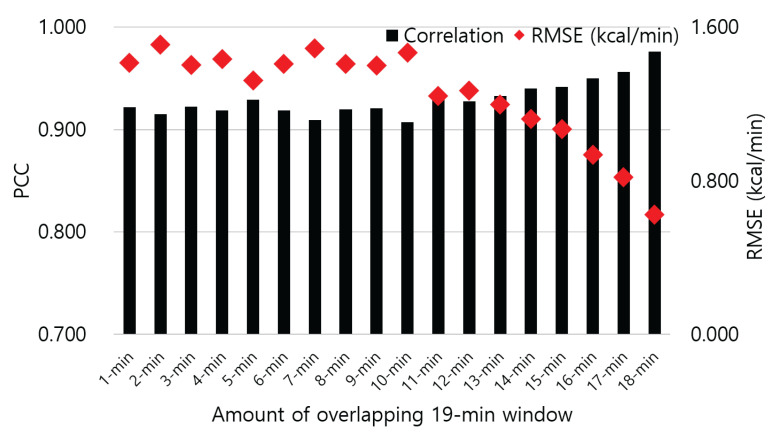
EE estimation results in terms of correlation (PCC) and error (RMSE) by using our proposed method with different overlapping sizes of 19-min window.

Figure 2 shows the highest PCC values for EE estimation across various window sizes. EE estimation using the M5 algorithm with 19-min window shows the highest PCC value (0.921) under RMSE of 1.406 kcal/min, which is the minimum among the entire values. Further, for all window sizes except 7 and 12 min, our proposed method shows higher PCC as compared to Conv. whose PCC is 0.829 and RMSE is 2.685 kcal/min. In terms of RMSE, our method was superior to Conv. for all size options of the window. Notably, both average and lowest PCC analyses also indicate that our proposed 19-min window EE estimation aligns most closely with the ground truth. In our 19-min window EE estimation, we adjusted the overlap, incrementing by 1 min, from 1 to 18 min. As shown in Figure 3, 19-min estimation overlapping 18 min of windows achieves the highest PCC, that is 0.976 (RMSE of 0.624 kcal/min). In this case, IBk algorithm is used for EE estimation model.

Table 6 shows correlation values and ranks of the entire features used in this study, calculated by using CorrelationAttributeEval in the Weka software (version 3.7.13). We averaged values of correlation and rank of each feature from estimation scenarios with windows ranging from 5 to 20 min. F_heart_ features generally rank higher and correlate more strongly than other features. According to average ranks, we determined combinations of 3 feature sets as F_heart_, F_heart_+F_exercise_, and F_heart_+F_exercise_+F_physical_. In case of a 19-min window with 18 min overlapping, IBk which results in the highest correlation using F_heart_+F_exercise_+F_physical_ (0.976) achieves correlations of 0.932 and 0.855 using F_heart_+F_exercise_ and F_heart_, respectively.

### 3.3. EPOC-Inclusive EE Estimation under Different Conditions

Table 7 presents the EE estimation results across various subdata conditions. We exploited the same estimation scenarios in Section 3.2 without overlapping windows. Each condition (each row of the table) corresponds to an estimation scenario of the highest PCC. Initially, while the optimal window size for EXER_all_ is 19 min, EXER_high_ and its subdata typically have smaller windows, such as 5 min for Male in EXER_high_. In contrast, EXER_low_ and its subdata maintain similar windows, like 18 min for Female in EXER_low_. Secondly, EE estimations for EXER_high_ and its subdata tend to outperform those for EXER_all_ in terms of PCC in general. Among those models, the highest PCC appears as 0.967 with RMSE of 1.043 kcal/min for the condition of BMI ≥ 23 in EXER_high_. Conversely, when compared to EXER_all_, the PCC values of EE estimation in EXER_low_ and its subdata are generally lower, with the exception of Female.

## 4. Discussion

This paper delves into EPOC-aware EE estimation through machine learning techniques. Based on our data from 32 participants, we crafted EE estimation models tailored to various exercise intensities and physical characteristics while adjusting design parameters of machine learning algorithms. Tailoring EE estimation to specific conditions appears to enhance the machine learning algorithm’s understanding of data characteristics. For example, EE estimation with 5-min window achieves the best performance in EXER_high_, while the one with 19-min window shows the best performance in EXER_low_. Indeed, high-intensity exercises elevate participants’ metabolic rates to a greater extent than low-intensity exercises [27]. Aggregating data over extended periods might obscure the dynamic shifts that occur in shorter time frames. Consequently, a more concise estimation window, such as 5 min for Male in EXER_high_, might be more suitable for high-intensity exercise data.

Compared to a broad estimation without categorization, the EE estimation for EXER_high_ data shows enhanced accuracy, whereas the estimation for EXER_low_ appears less accurate. To examine the reason of such degradation, we calculated PCC values between heart rate and EE within each experiment. Then, we averaged these PCC values within each data of Total, Male, Female, 10–20 s, 30–40 s, BMI ≥ 23, and BMI < 23. The average PCC values for all the conditions in EXER_high_ are higher than those in EXER_low_. Accordingly, we inferred that lower correlation between heart rate and EE in EXER_low_ results in lower estimation accuracy than EXER_high_.

Our method, employing a 19-min window with an 18-min overlap, yields the optimal EE estimation in terms of PCC. This estimation scenario produces the largest amount of data for training and testing compared to scenarios with less overlap. Machine learning techniques generally benefit from a larger amount of data to be trained. However, it is important to note that the volume of training and testing data does not always correlate directly with the estimation’s correlation coefficient. For example, the lowest correlation that is 0.907 appears in case of overlapping 10 min of the 19-min windows. Overlapping 10 min of the windows does generate larger amounts of data to be trained by machine learning algorithms than overlapping 1 min of the windows where PCC is 0.922.

While we successfully built upon the insights from our previous work [21] to improve the accuracy of EE estimation, we acknowledge that the scope of our study, with its 32 participants, may not be expansive enough to capture the full variability of the general population. The wide range of participant characteristics, such as age, height, weight, and BMI, as well as the inclusion of both genders, underscores the need for a larger sample size. A limited volume of data also raises two concerns in training and testing EE estimation models. Firstly, it constrained the categorization of our data into subdata in more detailed levels. For example, Male represents collected data from male participants who are ranged from 10 to 49 years with any BMI levels. If we categorize Male data into four subdata of ‘10–20 s and BMI ≥ 23’, ‘10–20 s and BMI < 23’, ‘30–40 s and BMI ≥ 23’, and ‘30–40 s and BMI < 23’, it could enable us to more customize EE estimation model. However, the size of our overall data is insufficient for such detailed categorization. For example, in EXER_high_, data of Male with the condition ‘30–40 s and BMI < 23’ is only one instance. Additionally, training models with limited data can result in overfitting issues [28]. It indicates that our designed model might not work accurately for the data collected from people who are not our participants.

Another constraint lies in the limited scope of exercise modalities and durations. We confined our measurements to walking and running activities, each conducted over a 20-min period. This limitation raises the question of whether our findings can be extrapolated to other forms of exercise and longer durations. Additionally, the study design did not include repeated measures for individual participants, which further limits our capacity to assess intra-individual variability and the repeatability of the data. The absence of repeated trials, combined with the constrained exercise types and durations, means that our study may not fully represent the complexities of EE estimation in more diverse and prolonged exercise scenarios. Moreover, we are unable to account for daily physiological fluctuations that could influence EE measurements, attributable to factors such as sleep quality, hydration status, dietary intake, and stress levels [29].

Despite these limitations, the present study lays an important groundwork for subsequent research. The data collected contributes valuable information to the field and highlights the necessity for more extensive research to develop robust EE estimation methods. The implications of our findings could extend to several health and fitness applications. Firstly, the enhanced accuracy of EE estimation derived from our EPOC-aware work has the potential to improve the measurement precision of existing fitness apps and services. By integrating our findings, these digital tools could offer users more reliable data, aiding them in better tracking their progress and making informed decisions about their health and fitness routines. Secondly, by factoring in the energy expenditure associated with different exercise intensities, our work could help in the prevention of overexertion during workouts. This has important implications for exercise safety, allowing individuals to avoid excessive durations or intensities that could lead to injury or other health issues [30]. Lastly, our research could contribute to the personalization of existing apps and services. By considering individual differences in EE, these platforms can tailor their recommendations more effectively, providing personalized workout plans and nutritional advice that align with each user’s unique physiological responses to exercise.

In upcoming studies, we plan to apply our method to a larger participant pool with broader contributing factors such as exercise type, exercise duration, race, smoking habit, medical history, and daily activity baseline. This would strengthen the statistical power and the representativeness of our results, affirming the validity of our data analysis with a more diverse population. Additionally, we will consider the integration of various sensors, like an electrodermal activity sensor combined with a heart rate monitor, to enhance the accuracy of EE estimation. In addition, we will evaluate diverse categorization criteria in EE estimation, including race, smoking habits, medical history, and daily activity baselines.

## 5. Conclusions

In this paper, we thoroughly studied EPOC-aware EE estimation using machine learning algorithms across different exercise intensities and physical attributes. From the data of 32 healthy participants, our EE estimation using a 19-min window yielded a PCC value of 0.921, comparing the estimated EE to the ground truth (RMSE of 1.406 kcal/min), while our previous work achieved a PCC value of 0.829 (RMSE of 2.685 kcal/min). Moreover, by categorizing our data based on exercise intensities and physical characteristics, our EE estimation model design achieved a PCC value of up to 0.976 (RMSE of 0.624 kcal/min). Our experimental results revealed that considering both EPOC and the context of exercise sessions (i.e., exercise intensities and physical characteristics) is significantly important in EE estimation.

## Figures and Tables

**Table 1:** Utilized features to estimate energy expenditure during and after conducting exercise.

Category	Feature	Description
F_heart_	HRmin	Minimum of heart rate values (bpm)
	HRp20	20% percentile of heart rate values (bpm)
	HRmed	Median heart rate values (bpm)
	HRp80	80% percentile of heart rate values (bpm)
	HRmax	Maximum of heart rate values (bpm)
	HRavg	Average of heart rate values (bpm)
	HRstd	Standard deviation of heart rate values
F_physical_	Gen	Gender of each participant
	Age	Age of each participant (year)
	Hei	Height of each participant (cm)
	Wei	Weight of each participant (kg)
	BMI	BMI of each participant, calculated by dividing
		weight with square of height (kg/m${}^{2}$)
F_exercise_	EX_T	Exercise duration (min)
	EP_T	EPOC-estimated duration (min)
	EX_int	Exercise intensity (40% or 70% of VO${}_{2}$ max)
	EX_or_EP	Indicator to represent whether unit data is
		collected during or after exercise

**Table 2:** Characteristics of study participants (N = 32; 11 females).

Characteristic (Unit)	Mean ± Standard Deviation	Range
Age (year)	28.2 ± 5.3	19–41
Height (cm)	170.9 ± 6.0	159.6–182.8
Weight (kg)	68.8 ± 8.9	53.7–92.4
BMI (kg/m${}^{2}$)	23.5 ± 2.2	19.0–28.8

**Table 3:** Average values for age, BMI, exercise duration, energy consumed during exercise (EE_exercise_), resting duration, and energy consumed during resting (EPOC) across 50 EXER_all_ experiments under different conditions (Exp.: the number of experiments).

Condition	Exp.	Age	BMI	Exercise	EE_exercise_	Resting	EPOC	EE_total_
				(min)	(kcal)	(min)	(kcal)	(kcal)
EXER_all_	50	28.8	23.5	20	148.8	26.7	60.4	209.2
EXER_high_	25	28.8	23.5	20	201.5	31.6	77.0	278.5
EXER_low_	25	28.8	23.5	20	96.1	21.9	43.9	140.0
Male	36	28.8	24.1	20	162.1	25.4	63.7	225.8
Female	14	29.0	22.2	20	114.4	30.1	52.0	166.4
10–20 s	28	24.4	23.2	20	162.3	29.5	65.0	227.3
30–40 s	22	34.5	24.0	20	131.5	23.2	54.6	186.1
BMI >= 23	28	29.4	24.7	20	153.1	24.8	61.0	214.1
BMI < 23	22	28.1	22.1	20	143.3	29.2	59.6	202.9

**Table 4:** Average values for age, BMI, exercise duration, energy consumed during exercise (EE_exercise_), resting duration, and energy consumed during resting (EPOC) across 25 EXER_high_ experiments under different conditions (Exp.: the number of experiments).

Condition	Exp.	Age	BMI	Exercise	EE_exercise_	Resting	EPOC	EE_total_
				(min)	(kcal)	(min)	(kcal)	(kcal)
EXER_high_	25	28.8	23.5	20	201.5	31.6	77.0	278.5
Male	18	28.8	24.1	20	218.2	29.3	79.6	297.8
Female	7	29.0	22.2	20	158.5	37.4	70.3	228.8
10–20 s	14	24.4	23.2	20	218.2	36.4	89.3	307.5
30–40 s	11	34.5	24.0	20	180.1	25.5	61.2	241.3
BMI >= 23	14	29.4	24.7	20	209.2	30.0	76.3	285.5
BMI < 23	11	28.1	22.1	20	191.6	33.6	77.8	269.4

**Table 5:** Average values for age, BMI, exercise duration, energy consumed during exercise (EE_exercise_), resting duration, and energy consumed during resting (EPOC) across 25 EXER_low_ experiments under different conditions (Exp.: the number of experiments).

Condition	Exp.	Age	BMI	Exercise	EE_exercise_	Resting	EPOC	EE_total_
				(min)	(kcal)	(min)	(kcal)	(kcal)
EXER_low_	25	28.8	23.5	20	96.1	21.9	43.9	140.0
Male	18	28.8	24.1	20	106.1	21.5	47.8	153.9
Female	7	29.0	22.2	20	70.3	22.9	33.7	104.0
10–20 s	14	24.4	23.2	20	106.4	22.7	40.6	147.0
30–40 s	11	34.5	24.0	20	83.0	20.8	48.0	131.0
BMI >= 23	14	29.4	24.7	20	97.0	19.6	45.7	142.7
BMI < 23	11	28.1	22.1	20	94.9	24.8	41.4	136.3

**Table 6:** Correlation coefficient values and ranks of features for EE estimation, calculated by using CorrelationAttributeEval in the Weka software [23] and averaged across different window sizes.

Category	Feature	Average Correlation	Average Rank
F_heart_	HR_min	0.296	7.9
	HR_p20	0.604	3.9
	HR_med	0.658	1.6
	HR_p80	0.652	2.1
	HR_max	0.493	5.1
	HR_avg	0.646	2.4
	HR_std	0.193	8.2
F_physical_	Gender	0.168	8.7
	Age	−0.052	13.8
	Height	−0.028	13.2
	Weight	0.091	11.0
	BMI	0.149	9.4
F_exercise_	ExerDuration	0.087	10.9
	PostDuration	−0.133	14.9
	Intensity	0.253	6.8
	Exer_or_EPOC	−0.655	16.0

**Table 7:** EE estimation results in case of the highest PCC value for different conditions (without overlapping windows).

Condition		Machine Learning Algorithm	Winow Size	PCC	RMSE
EXER_all_	Total	M5	19 min	0.921	1.406
EXER_high_	Total	IBk	6 min	0.958	1.228
	Male	IBk	5 min	0.966	1.253
	Female	MLPR	16 min	0.957	0.870
	10–20 s	IBk	5 min	0.962	1.275
	30–40 s	LR	9 min	0.953	1.125
	BMI ≥ 23	AR	16 min	0.967	1.043
	BMI < 23	IBk	5 min	0.960	1.215
EXER_low_	Total	LR	19 min	0.791	1.287
	Male	MLPR	19 min	0.804	1.391
	Female	LWL	18 min	0.937	0.314
	10–20 s	IBk	20 min	0.848	1.234
	30–40 s	DT	19 min	0.729	1.062
	BMI ≥ 23	IBk	20 min	0.711	1.469
	BMI < 23	MLPR	19 min	0.874	1.021

## Data Availability

Data are contained within the article.

## Abbreviations

The following abbreviations are used in this manuscript:

EPOC	Excess post-exercise oxygen consumption
RMSE	Root mean square error
EE	Energy expenditure
PRE	Preparation stage in the experiment
EXER_low_	Low-intensity exercise stage (40% of VO${}_{2}$ max in the experiment) in the experiment
EXER_high_	High-intensity exercise stage (70% of VO${}_{2}$ max in the experiment) in the experiment
REST	Resting stage with the least movements in the experiment
EXER_all_	The entire exercise experiments
VO${}_{2}$	Maximal oxygen uptake
BMI	Body mass index
HR	Heart rate
Conv.	A conventional method of EE estimation
PCC	Pearson Correlation Coefficient
LR	LinearRegression
MLPR	MLPRegressor
AR	AdditiveRegression
DT	DecisionTable
M5	M5Rules
